# Dr. Beddoes's Collection of Papers on the Influenza

**Published:** 1803-10-01

**Authors:** 


### Medical and Physical Journal.

VOL. X.] OCTOBER 1, 1803. [NO. LVI.

*Printed for R. PHILLIPS, by W. Thorne, Red Lion Court, Fleet Street, London.*

### DR. BEDDOES'S COLLECTION OF PAPERS ON THE INFLUENZA.

### PART II. continued.

### 72. Mr. WILLIAMS, Merthyr, Glamorganshire, June 7, 1803.

NO facts have occurred to me to enable me to form a *decisive* opinion, whether the influenza is infectious or otherwise; but I am inclined to think that it is contagious, for the following reasons: Whenever one in a family was attacked with it, in general the major part or the whole of that family caught it; the great and sudden depression which so constantly attended it, bears strong affinity to other contagious diseases, and also its general progress. The disease appeared at Surowy iron-works, eight miles from Merthyr, about the 20th of February, and in a few days after at Merthyr, and it has not yet entirely disappeared. I did not observe that the men were attacked in sets or classes at Merthyr iron-works or the neighbouring iron-works. This is the sum of what I have had opportunities of remarking.

### 73. Dr. HOBBES, Swansea, April 15, 1803.

The few observations I have made respecting the influenza, lead me to judge that it is contagious.

I was induced to form this opinion from remarking that the influenza had travelled (if I may be allowed to use the expression) from the east to this place. For we heard of a complaint which attacked whole families in the neighbourhood of Cowbridge, Pyle, and between Pyle and Briton Ferry, before the influenza was known here. I have also been credibly informed, that the influenza did not reach that part of Carmarthenshire which is near to this place, until after it had made its appearance at Swansea. The following fact likewise disposed me to consider the complaint as contagious: A gentleman and lady came in the beginning of March, upon a visit to a gentleman's house in the neighbourhood of Briton Ferry, where some of the family were labouring under the influenza. They came from a part of Carmarthenshire where the disease had not shewed itself. The lady was seized in the course of a few days after her arrival, and the gentleman a few days afterwards.

On comparing a number of cases, I do think the influenza distinguishable from common catarrh. I cannot, however, myself, call to mind a case (although other medical men seem to think they have seen such) where catarrhal symptoms of some description or other did not take place. But they could not, in my opinion, determine the disorder to be merely common catarrh. For example, a lady was teased with incessant cough, which lasted from four o'clock in the evening until four o'clock the next morning, it then left her; she had no more of it, nor had she any other symptom of catarrh; fever however continued with pain of the back for some days. Others have been seized with running at the nose, which lasted several hours without any other catarrhal symptom; fever, with pain of the back, then succeeded, and lasted some days.

In those instances, where the chest was much affected, and symptoms of pneumonia took place (which mostly happened when people advanced in years were attacked) it might be doubtful whether the symptoms belonged to common catarrh or the influenza.

The depression in this complaint, is certainly greater and more sudden than is ever observed in common catarrh. Indeed, the depression has always appeared to me a distinguishable and leading symptom of the complaint. I have seen the disorder gradually proceed to the most formidable and fatal stage of pneumonia, and I have now a case where it has ended in low fever with extreme debility, but without any affection of the chest whatever.

The first case of well-marked influenza, I think, I saw on the 7th of March. The last case, on the 10th of April.

Last week I heard of the whole of a large family in the country being recently attacked. I do not hear this week of many fresh seizures.

74. Mr.

74. Mr. Harness, Tavistock, July, 1803.

The influenza first appeared in this neighbourhood about the middle of March, and went off the latter end of May.

It appeared in the town three weeks earlier than in the adjacent county, except in one instance.

After being general, *I* did not meet with *single* instances.

It certainly seemed to *me* to pass from person to person for the following reasons.

Two ladies of this place spent a few days at Exeter, and slept at a friend's house, where the family had been ill of the influenza (and indeed some part of it then laboured under the complaint): one of them was seized as she was returning, and the other two days after, and the whole of the family where they lodged had the complaint within ten days. About the same time, a person coming from Plymouth Dock, (where the influenza was very prevalent) was seized at a friend's house at a different part of the town from the ladies just mentioned, the family of this house likewise soon became infected; these were the first instances of the complaint in this town, but it soon became general.

I have not had any reason to suspect the contagion conveyed by articles of dress.

Several cases have occurred of a violent ardor urinæ (in women more particularly), this however soon gave way to the usual medicine.

Postscript from D. Geddes, Esq.

Mr. Harness desires me to add, that Mr. Carpenter's coachman returned very ill of the influenza from Bath, nearly about the same time, and communicated the disorder to his family, who live a mile from the town.

75. Dr. Vaughan May, Plymouth Dock, June 7, 1803.

During the months of November and December last, the winds prevailed in the eastern quarter, which continued in January and part of February of the present year. In this period, diarrhœa and cholera were very prevalent; so nearly similar to that preceding the influenza of 1788, that to many of my friends, I hazarded a pretty confident opinion of an expected return. In this I was not deceived, for about the latter end of February, it began its course. About the middle of March, I believe it was most prevalent, at least I found it so; and the last well-marked case I was called to on the 30th of April.

The symptoms were nearly similar to those I described in 1788, but I observed the debility in general was more considerable. Pneumonia in some cases prevailed so as to threaten danger, but I met with no loss of patients, nor did any of my medical brethren, excepting in a few instances of advanced age. Those who were attacked, excepting as above, recovered in proportion to their early or late applications for assistance; and I found no difficulty in speedily removing their complaints, by vomiting with tart. antim. & ipecac. which in general, if exhibited on the first or second day, totally removed every complaint. In other cases antimonial powder in small doses, combined with opiates and diluents, proved highly useful. In no case did I find it necessary to bleed; for though, in many instances, pneumonia was carried to a great height, yet, in my opinion, the pulse and general and excessive debility forbade it. In some few cases, after the fever had left the patients, and they were again about, I met with a violent, dry, spasmodic cough, which did not give way to opiates; but when combined with digitalis in the dose of a grain of each night and morning, it completely answered my most sanguine wishes, after a very few doses.

Blistering was had recourse to in some instances, and I think with advantage; more particularly so, in those cases resembling low fever, of which there were many; and here I found the greatest advantage from bark and wine, after premising an emetic and laxative.

I am inclined to consider the influenza contagious, for when it first made its appearance, it certainly attacked individuals; and I have observed many instances where the children in families were first affected, and afterwards their parents; and in other instances, vice versa.

It certainly was no uncommon thing for many o a family to be seized together, and this has operated on the minds of many against contagion, and in favour of diffusion of pestilence through the atmosphere. Has this not been the case with the epidemic measles and small-pox, as described by Sydenham and others?

I know but of few exceptions where it has entered a family, and there was certainly no difference from exceptions in measles, &c. In many instances, undoubtedly, the resemblance to common catarrh was such as to make the decision doubtful. I think the only symptom which decided the complaint in such cases, was debility, and it certainly did appear in all gradations from pneumonia to low fever.

I never observed that any particular classes were exempt, but but the soldiers in the ordnance department were certainly not so generally affected as in the influenza of 1788.

It seems to me there was pretty much regularity in the rout of the disease. I believe there can be no doubt of this being the same with that of Paris, and which we have understood carried off such a number of people. From Paris it broke out in London, taking a western course until it reached this place; from hence it went into Cornwall, where it now rages, as well as in Ireland. This has been urged to me in favour of diffusion of pestilence through the atmosphere! In that case, how is it that it did not prevail in two neighbouring counties, viz. Devon and Cornwall, at the same time? which it certainly did not. This appears to me a strong point in favour of contagion.

As this is a matter of some importance, I shall be happy to see your opinion at large on the subject; and if these observations are of any use to you, they are quite at your service.

76. Mr. Whitlock, Ottery St. Mary, July 15, 1803.

I attended many patients in the latter part of the month of February, whose symptoms were not sufficiently marked for me to say decidedly whether they were cases of common catarrh or influenza. In the beginning of March, however, the characteristic symptoms of influenza were too apparent to be mistaken, and its distinctive character was never completely lost until its total disappearance, which, in my practice, took place about the first week in June.

The date of its commencement *was* different in different situations. I did not attend any patient out of the parish of Ottery, until the end of March.

I attended but few *solitary* cases during the time of its being rife in this neighbourhood, and those *solely* in the early part of its progress.

As far as my observations go, the disease was most decidedly *contagious,* and *readily* communicable from one person to another. Many had second attacks in consequence of their attendance on sick relatives.

I will take the liberty of adding, that very young infants appeared less susceptible of the infection than adults; nor did I see one case where they did not resist the disease altogether, until many of the persons, to whose care they were committed, had been attacked by the complaint in succession.

77. Mr. Gidely, Penzance, July 10, 1803.

The influenza began first in the parishes nearest the Land's End, about the beginning of March, and was prevalent there at least a week or ten days before its appearance in this town, which was on the 25th of March, and then it was general over the whole neighbourhood, and did not cease to be so until the middle or latter end of April. Single instances occurred for a long time after, and cases till very lately have appeared in different places.

I thought it evidently infectious, and Mr. Borlace says he has no doubt of its being so, having marked the progress of it in his own family, himself having caught it during his visiting some patients confined with it, and those who were most with him following in succession.

Whether conveyed by articles of dress, I cannot answer, as I never observed it.

A family now residing here, but who belong to Essex, and for particular reasons had to dread the disease, used every means of prevention, as avoiding communication or converse with infected houses, wearing camphor, using vinegar, &c. and were fortunate enough all to escape. I believe it was the only house where some or all of the family were not attacked; not one medical man escaped.

It was mortal only in a few instances, and the subject was either consumptive or asthmatic, and its course to a fatal termination was then very rapid.

The disease appeared at Helston about a fortnight after its being general here, but was so slight a disease that the surgeons can give no account of it.

78. Mr. Williams, Aberystwyth, July 8, 1803.

The influenza appeared here about the 17th or 18th of March, exerted its greatest influence from the 1st to the 13th of April, and did not entirely leave us till the second week in May.

It appeared in different places at different dates. My practice takes in a circuit of about twenty miles. It was known twenty miles south of this place, ten or twelve days before it was here, and was upwards of a fortnight before it extended the same distance north.

The symptoms were nearly the same in all, but much more severe in those who had asthmatic affections; it proved fatal in this neighbourhood in one instance, and that was an elderly lady who had for some years been subject to severe paroxyms of asthma.

I have

I have some reason to think, that persons of a scrophulous consumptive habit, and who were liable to hæmoptysis, escaped this epidemic. If such is the fact, it cannot have escaped Dr. B's observation, and I shall be glad to know it.

I always thought it contagious, and have had no reasons to think otherwise.

### 79. Dr. Black, Newry, May 28, 1803.

I have seen a great deal of the influenza for more than two months past, and at no time have I entertained a doubt of its contagious nature. In general, it diffused itself through all the members of a family in which it once got a footing; and though many negative instances might be quoted, yet I am very far from thinking that these ought to overturn the affirmative proposition obviously resulting from a full induction of particulars. I do firmly believe that the negative examples in the most highly contagious diseases, of which we have any knowledge, plague for instance, and small-pox, are to the full as numerous and as remarkable as they have been in influenza. As far as I have been able to learn, its first appearance in this kingdom was in Dublin, the latter end of February or beginning of March, whence it gradually and not very rapidly diffused itself northwards, until it reached us the beginning of April, and so on in a regular progress. I will mention to you one circumstance which, I think, well illustrates its slow propagation from one district to another in the ordinary way of contagious diseases. At the time that the disease was raging at its height in this town, when scarcely a family was free from it, my friend, Dr. Crawford (brother to the late Adair Crawford) who resides at Lisburn, a town twenty-three miles north of this, was, on the 23d of April, called hither to my assistance in a case of another kind. He was much surprized when I informed him how very general this disease was with us, and how severe. He said, at Lisburn they were, *as yet*, unacquainted with it. But I understand that the town and neighbourhood of Lisburn have since that time suffered as much as other districts.

There was a circumstance observable in the nature of the disease itself, which appeared to me to convey strong evidence of its contagious nature. In a great number of instances, when the patient on the first seizure took a brisk purge of calomel and scammony, and instantly after that had operated, a sudorific medicine, he was free from all fever on the third day. Yet, under these circumstances, there remained often for a week or longer, a debility, lassitude, prostration of strength and spirits, and loathing of animal food, which I was utterly at a loss to account for, except on the principle that a febrile contagion had been introduced into the system, and though its virulence or activity was mitigated or subdued, yet the effects enumerated (which are its ordinary effects) remained for some time.

It was very mortal in this country both in the higher and the lower classes, but on a different principle in these respectively. My opportunities of observation among the lower classes were not numerous; but I had reason to be satisfied that one great cause of its fatality was improper treatment, and more especially a liberal use of their panacea, whiskey In the better ranks, the chief sufferers were persons advanced in years, of full habits, and who had any weakness of the lungs, or afflux of fluids to that viscus, and more especially those who had been free livers. In many such the disease terminated by an effusion into the bronchiæ, for the elimination of which, the powers of expectoration were inadequate. I observed that emetics had a favourable effect on the expectoration, and opiates a most pernicious one

80. Dr. Ryan, Kilkenny, July 1803.

The influenza, which has so lately extended its influence all over the united kingdom, first made its appearance in this city the beginning of April last. Before it reached this quarter, or as far as I can learn, any other interior part of the country, it raged with great violence for many days in the principal sea port towns, as in Dublin, Cork, and Waterford; so that the probability is, it was imported on board of ship by passengers from England, Scotland, or some other country trading with us.

The first intimation I had of its approach was in the following way. A gentleman residing about eight miles from this place, on the high road from Cork, came to consult me about the state of his health, and during our conversation, thought it incumbent on him to inform me that he and his whole family had just recovered from the influenza. The day immediately preceding this, I was called upon to visit a patient, whom I considered to labour under a bilious fever, attended by a catarrhal affection, (a very usual combination in this part of the country) and treated it accordingly.

About

About the fourth day the febrile symptoms being evidently on the retreat, while the cough continued more troublesome than before, I began to reflect on the conversation I had with my patient; and taking into consideration the circumstance of the influenza, (according to the reports of the newspapers) having prevailed in Cork for three weeks past, I now ventured to pronounce, that what I took for a bilious fever was in reality the influenza, which had got footing in different parts of this island. The event justified my opinion; the disorder, in less than a fortnight, having spread itself all over this city.

In some country places, isolated and detached from any adjacent dwelling, I have known the whole family continue exempt from the influenza until one of them happened to come to town, bring it home, and after a few days confinement, communicate it to the remainder. Though this epidemic diffused itself very extensively through all ranks and descriptions of people, I have, notwithstanding, observed many entire families to escape, that must have been constantly exposed to infection, supposing it to exist; nay, I have remarked that some who acted in quality of nurse-tenders in the family way, did not take it; but these were few indeed, when compared with the immense numbers who caught it by their attendance on the sick.

One source of error on this subject ought not to be passed by unnoticed; sometimes the symptoms were so slight, trivial, and evanescent, that many supposed they were fortunate enough to have escaped, who, in fact, had passed through the ordeal, but in such a way as scarcely to be conscious of any indisposition. From all this you will readily perceive that I am among the number of those who suppose the influenza to be contagious; and I really am so, but the limits of this letter prevent me from investigating the subject more in detail, and enumerating the various motives that determine me to this belief.

Before I enter upon a description of the disease in question, you may not probably think it irrelevant to the present enquiry, that I should transcribe, verbatim, from my book of reports, a note which I preserved of an epidemic catarrh, that prevailed very generally here in December, 1800.

*Kilkenny, Jan.* 1, 1801.

An epidemic catarrh or influenza has been very prevalent in this city and its neighbourhood for three weeks past and and more, and was ushered in with the usual symptoms of pains in the limbs, a sense of cold or chilliness, cough, and coryza, with more or less of fever. In many instances, a considerable degree of inflammation of the lungs accompanied this disorder, which required copious bleeding and blistering, much more, indeed, than I ever found necessary in any similar epidemic. While this complaint was at its height, several cases of strongly marked pleuritis occurred at the same time. Whether they originated altogether in the reigning epidemic or no, it was not an easy matter at times to determine, but on some occasions such a connection was apparent; for though the characteristic symptoms of pleuritis were all present, still the softness of the pulse clearly pointed out a difference between it and the common pleurisy, to be met with every day. Moreover, the former did not by any means bear a repetition of blood-letting, as well as the latter usually does, except a relapse was brought on by exposing the body too soon after recovery to cold air, when it became necessary to use the lancet with as much freedom as in any other case whatsoever. In general the complaint was of a mild nature, and for the most part required no medical aid; abstaining from animal food and wine for a few days, and lying a bed were sufficient to accomplish a cure.——But to return to the immediate subject of my letter.

Children were not so liable to be attacked as grown-up persons, and with the former it was comparatively a mild disease; I saw but two children seriously ill, and they were relieved by the application of blisters between the shoulders, and a plentiful use of squills. Some, after having been inoculated with the vaccine pock, caught the influenza; but neither complaint seemed to have been aggravated by the complication.

The epidemic, as it occurred in this city, may be properly enough divided into two varieties; the one a very mild, though often a tedious disease; the other a highly distressing, and sometimes a formidable malady. The former resembled in no small degree the common and usual effects of change of temperature on the human body; but the latter, particularly in its aggravated form, was, toto cælo, a different complaint, and had nothing in common with catarrh, except the cough and coryza. In the one, lassitude, pains in the joints and muscles, frequent cough, unusual sensibility to cold, and some slight degree of a febrile state, were the predominant symptoms, but not so intense as to necessitate the patient's confinement to bed: in the other, the the paroxysm of fever was similar to that of synochus or typhus, and could not be distinguished from them at the first onset. The patient, after a shivering fit, complained of sickness at stomach, frequently vomited, and threw up some bilious sordes, the bowels at the same time being attacked with a dysentric kind of purging, though sometimes they suffered severely from pain without any accompanying evacuation.

A cough seemed to be a necessary constituent part of the disease, and was seldom absent; and next to it in frequency of occurrence, were a severe pain in the forehead and soreness of the throat; bleeding from the nose was by no means rare, and I met with one case of it which endandered the life of the patient.

In the accounts of this epidemic already published, some have considered it as connected with debility, and others with increased systematic excitement. The truth lies in the middle between these two opposite extremes. The pulse was in general rather full, soft, and easily compressible, nearly similar to that of the bilious fever of this country in its earlier stage; I have witnessed many protracted cases without any striking mark of debility, and no tendency to putrefaction. On the other hand, it could not, in strictness of medical language, be denominated an inflammatory disease, as no instance of it whatever, unless mismanaged, in all my practice, demanded venestceion; it is but fair, however, to acknowledge, that when appearances became alarming, they were very generally the consequence of an inflammatory affection of the lungs, superinduced by a heating and improper regimen, or by an unseasonable exposure of the body to cold, before recovery was complete. On these occasions the blood threw up as large a portion of coagulable lymph, and was as completely cupped as in genuine cases of pneumonia or pleuritis. The fever attending the influenza most commonly began to subside about the fourth or fifth day, but in many cases it was lengthened out to a period of seven, nine, or twelve days; and in this continued form, a delirium of the phrenitic kind was occasionally to be met with, which excited no small degree of interest and alarm.

In a small town, named Callan, within eight miles of this city, most of those who were attacked with the influenza had an eruption so exactly resembling the scarlatina, that it became a difficult matter to distinguish the one from the other, particularly from this circumstance, that an inflammatory affection of the uvula and velum pendulum palati was a constant attendant on this variety of the epidemic. An intelligent apothecary of the place, from the observations he had made, considered the eruption a critical effect, as he found that an evident change for the better was perceptible immediately on the eruptive matter being thrown out on the surface. I interrogated him minutely as to the existence of any other epidemic, more especially the scarlatina anginosa, and he positively affirmed that no such disorder as this had shewed itself in the town or neighbourhood thereof for several months past, but from the first appearance of the influenza he had met with cases of measles, and that it has from that period to this prevailed very generally all over the district.

The first and most essential step in the cure, according to my observations, was clearing the stomach and bowels by an emetic, and some gentle laxative medicine; after this it was usual with me to administer small doses of James's powder, washed down with a spoonful or two of the ammonia acetata, or a solution of some other neutral salt, every four or six hours, until some irritation took place in the stomach or bowels, or symptoms of convalescence began to appear. The urgency of coughing, and labour of respiration being so frequently combined, at first rendered me doubtful whether or no I should fly for relief to the use of the lancet. But a little time, and further acquaintance with the nature of the epidemic, tranquilised my mind on this point, and obliged me to abandon every idea of blood-letting, except in particular cases, where, from the previous condition of the lungs, or an ill-judged and unappropriate use of stimulants, such a measure had become necessary and unavoidable. In three or four instances of this kind, the utility of venesection was unquestionable, and the blood (as before observed) was equally buffy and cupped, as in genuine cases of inflammation of the lungs. Blistering between the shoulders was the remedy in general resorted to, when the breathing was oppressed and difficult, and it was found decidedly beneficial in removing this symptom, as well as the delirium which was constantly found to accompany it. When the delirium continued obstinate, blisters were applied to the legs and other parts, and the head was shaved and bathed with cold water and vinegar; but if these likewise were found to fail, recourse was had to laudanum, and most commonly with very signal success.

One of the most striking peculiarities attending this complaint, was the slowness with which almost every person, son, who was severely attacked, advanced towards recovery, the functions of the stomach continuing in a state of derangement, with symptoms of dyspepsia and hypochondriasis, long after the disappearance of the fever. The pulmonary organs in like manner remained a length of time delicate and irritable, suffering from the most trivial irregularity, or any sudden change of temperature in the atmosphere. There cannot be a doubt but it has laid the foundation of phthisis pulmonalis in many habits, particularly in those predisposed to this disorder. I have under my care this moment, three cases of it in the incipient stage, evidently originating in this source, and not the slightest tendency to pulmonary consumption appeared in any one of them previous to the appearance of the influenza.

81. Dr. Halliday, Belfast, May 30, 1803.

The influenza has been so slight here that physicians have not been much applied to on account of it. My own observations are too limited to be worth detailing, but sufficient to convince me, that however the disease may in some instances depend on the influence of the atmosphere, yet in the greatest number of cases I have met with, it could only be accounted for by the supposition of contagion. It prevailed very generally in Dublin several weeks before it appeared here. The first case we had of it, (as far as I can learn), was in a house, in which a Dublin gentleman was on a visit; two or three days after, two or three of the family became affected. Its progress in one house in the country, where I attended, was remarkable. Mr. P. and his family were first attacked, then the female servants, and after them the men servants; of twenty-one in that house three only escaped it. In another house, not far from that, not one has had the disease. In Belfast many houses have escaped; whilst, in many others, not one of the inhabitants has escaped. The medical men here have, in general, been exempt. In my own house it has not affected an individual.

82. Mr. H. Edgeworth, Edgeworth Town, June 19, 1803.

It was the expectation of hearing from Dublin that prevented me from writing sooner; but as such delay may be inconvenient, I send you the opinion of Mr. Dubourdieu, surgeon to the infirmary in this county.

He

He distinguishes the influenza from a common catarrh, by the suddenness with which a person is seized, the violent pains in the calves of the legs, muscular parts of the thighs, between the shoulders and back of the neck; pulse full, and difficulty of breathing very great. Great bleeding sunk the pulse considerably, but the breathing became more oppressed, and considerable debility was occasioned, which brought on delirium and often death, an instance of which happened yesterday; notwithstanding this, very gentle bleeding has been found of service.

All those who went to bed were seized with perspiration for twenty-four hours.

The influenza appeared here about three months ago; it appeared some time in Dublin before this period.

Although the air seemed infected with contagion, Dubourdieu is convinced that the influenza was communicated from one person to another. I have the same opinion from another practitioner in this neighbourhood.

It appears, as I suppose you have constantly observed, that those who died of the influenza, were those who were old and infirm, or the very young; and lastly, those who had some previous disorder.

The influenza is still prevalent in this country, particularly amongst the poor. I am happy to give you an instance, where the influenza was brought from Dublin by parcels, which were made up by a person very ill of that disorder, and sent to a lady of quality here. As soon as the parcels were unpacked, the person who unpacked them was first seized, then the lady herself, and the whole family were infected, as was the neighbourhood. The influenza seizes people more than once. I know of an instance where a person had it three times.

In a subsequent letter, dated Callan, July 19, Mr. Henry Edgeworth makes the following reply to my inquiries concerning the authenticity of the fact, related at the close of his first letter.

Respecting the influenza being conveyed by articles of apparel, I cannot vouch more for the authenticity of the fact, than that it was communicated by a letter from the lady herself, who lives nine miles from us, and also that it was the opinion of Mr. Dubourdieu, the surgeon, that it was from those parcels the persons who unpacked them received the infection.

